# Non-ketotic Hyperglycemia Hemichorea-Hemiballismus Syndrome: A Case Report

**DOI:** 10.7759/cureus.38434

**Published:** 2023-05-02

**Authors:** Scott Everett, Alice M Dalo, Deepasri Ananth, Andrew L Alejo, Haley Durdella, Matthew Niehaus

**Affiliations:** 1 College of Medicine, Northeast Ohio Medical University, Rootstown, USA; 2 Department of Emergency Medicine, University Hospitals Cleveland Medical Center, Cleveland, USA

**Keywords:** gaba, choreiform, hyperglycemia, case report, non-ketotic hyperglycemia hemichorea-hemiballismus syndrome

## Abstract

Non-ketotic hyperglycemia is an uncommon cause of hemichorea-hemiballismus syndrome that has been associated with high levels of glucose that are not well controlled. Lesions typically occur in the globus pallidus and putamen, which can be identified via computed tomography (CT) or magnetic resonance imaging (MRI). These lesions generally correspond with ballistic and choreiform movements on the contralateral side of the observed imaging findings. Additionally, amelioration of hyperglycemia is the first-line treatment and usually reduces and resolves these hyperkinetic movement symptoms. This case report demonstrates a case of non-ketotic hyperglycemia hemichorea-hemiballismus syndrome in an individual with a history of poorly controlled type 2 diabetes mellitus and a highly elevated hemoglobin A1C (HbA1C), who subsequently improved with insulin therapy.

## Introduction

Non-ketotic hyperglycemia hemichorea-hemiballismus syndrome is a rare and uncommon disease where a non-ketotic hyperglycemic state causes the hyperkinetic movements commonly seen in neurodegenerative disease including basal ganglia degeneration. Patients initially present in a severely hyperglycemic state with no evidence of diabetic ketoacidosis and have uncontrollable movements of the upper or lower extremities as well as sometimes the trunk. The pathophysiologic mechanism is not fully understood, but poor glycemic control in the putamen is found in almost all patients with the disease. To our knowledge, there have been two published case reports of this presentation in males with a history of diabetes. We present the case of a 68-year-old male who presented with choreiform movements of the upper extremity secondary to non-ketotic hyperglycemia that decreased in severity with insulin treatment to return the patient to a euglycemic state.

## Case presentation

A 68-year-old male with a past medical history of poorly controlled type 2 diabetes mellitus presented to the emergency department (ED) with uncontrolled movements of the right upper extremity that had begun spontaneously three days prior to presentation. These movements were initially limited to the right hand but progressed to involve the entire right upper extremity. He reported associated right upper extremity pain but was otherwise asymptomatic. He denied prior episodes of these spontaneous movements, as well as any recent history of viral illness or trauma. He also denied other symptoms including chest pain, headache, cough, fever, and chills, and his vitals were within normal limits.

**Figure 1:**
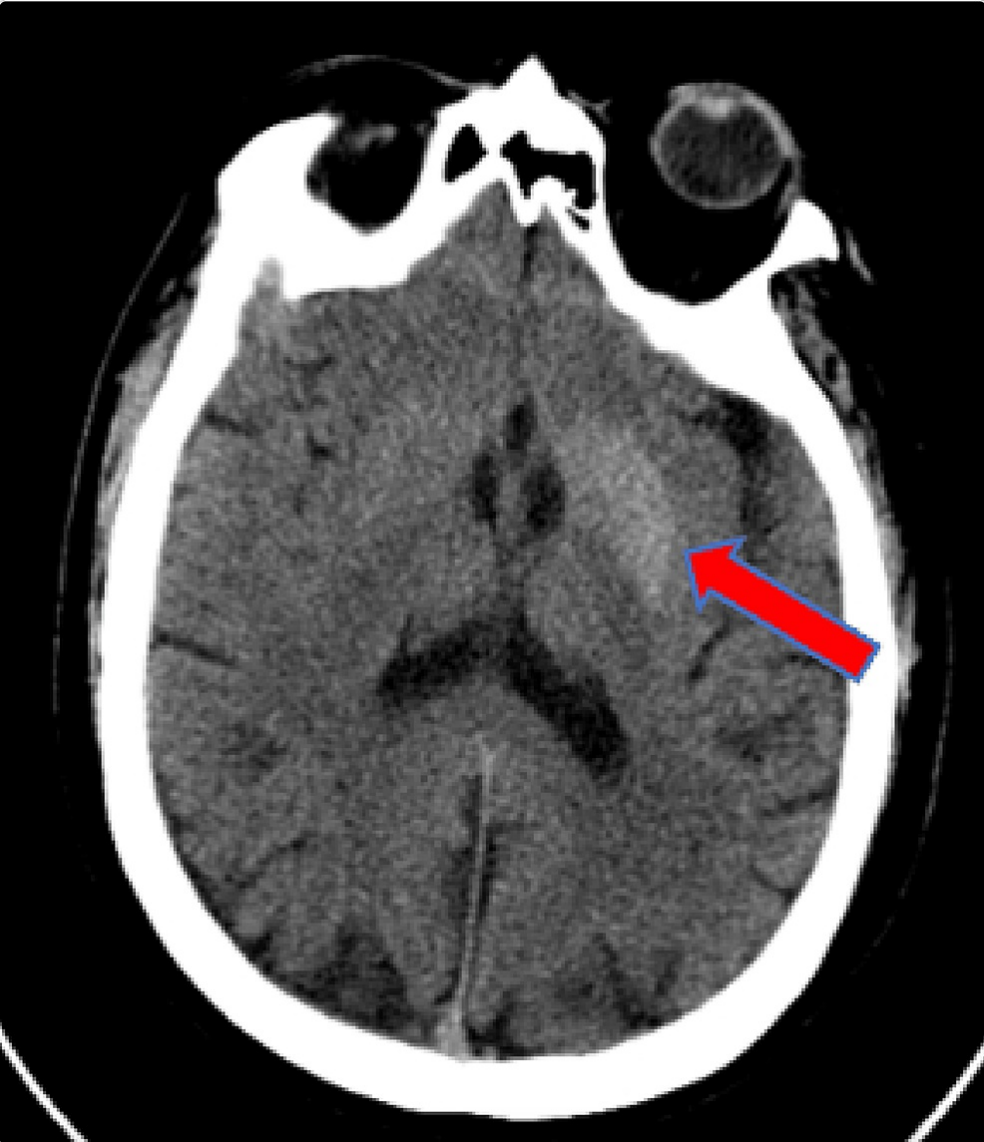
Head CT without contrast Hypodensity in the left putamen and globus pallidus (arrow) CT: computed tomography

**Figure 2:**
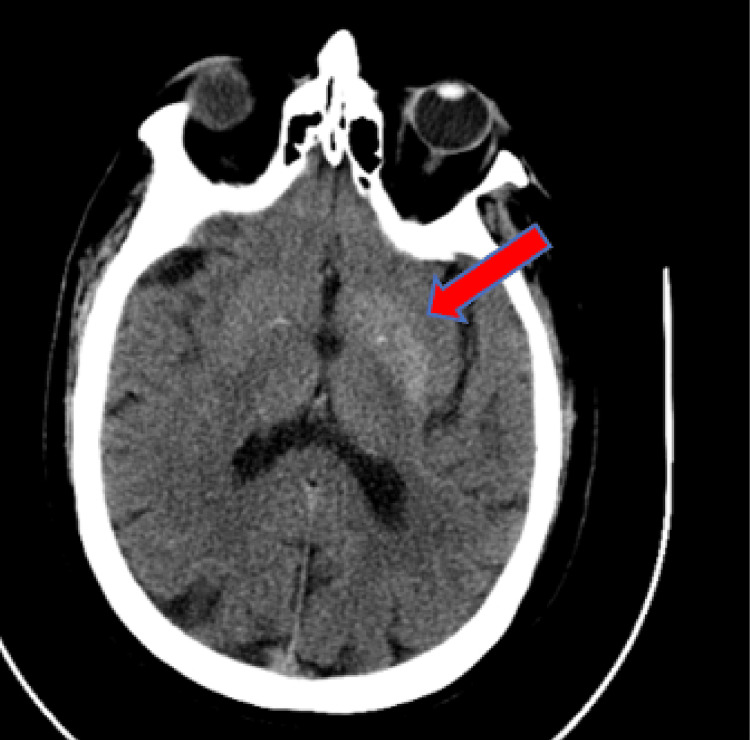
Head CT without contrast (subsequent slice) Hypodensity in the left putamen and globus pallidus (arrow) CT: computed tomography

**Figure 3:**
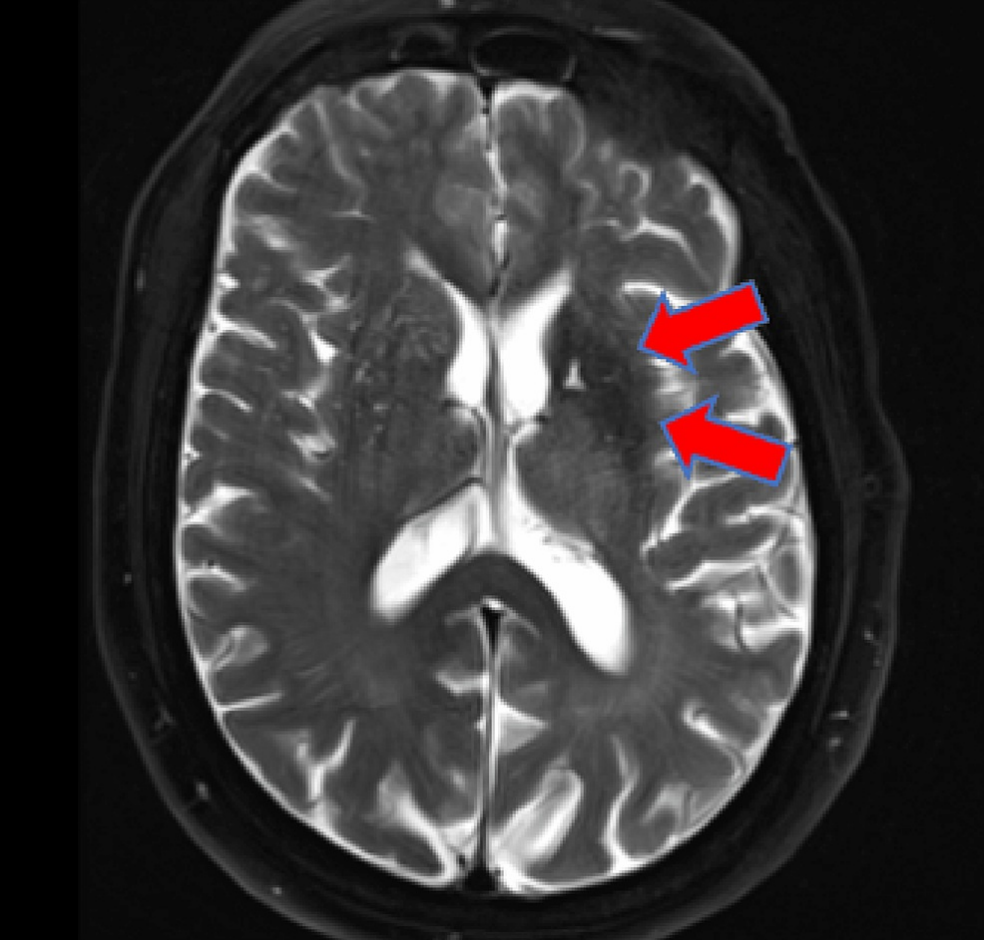
T2-weighted FLAIR MRI Hypointensity in the left lentiform nucleus (arrows) FLAIR: fluid-attenuated inversion recovery, MRI: magnetic resonance imaging

On physical examination, the patient was noted to have writhing movement of the entire right upper extremity. The movement could be voluntarily stopped with muscle contraction, but the patient reported that this would elicit severe pain from the shoulder radiating to the hand. Decreased sensation to light touch was noted in the bilateral lower extremities extending circumferentially from the feet to the knees. The patient displayed full and symmetric strength in the extremities. No deficits were noted on cranial nerve testing, and the patient demonstrated no gait abnormalities. The remainder of his examination was unremarkable. The patient’s initial laboratory testing was notable for elevated serum glucose of 423 mg/dL, blood urea nitrogen of 30 mg/dL, and creatinine of 1.52 mg/dL. Due to persistent neuromuscular symptoms, a non-contrast head computed tomography (CT) and head magnetic resonance imaging (MRI) were obtained to look for possible causes of the patient’s symptoms. The CT showed a subtle hypodensity in the left putamen and globus pallidus (Figures 1, 2). T2-weighted fluid-attenuated inversion recovery (FLAIR) MRI showed hypointensity of the lentiform nucleus on the left side (Figure 3).

Neurology was consulted and felt that the patient’s symptoms and imaging findings were consistent with non-ketotic hyperglycemia hemichorea-hemiballismus syndrome. The patient was initially treated with 15 units of regular insulin and six units of Lispro while in the emergency department. He was then admitted to the internal medicine service for further evaluation.

During his hospital course, the patient’s insulin regimen was adjusted, and symptoms were also treated with risperidone. Serum glucose remained elevated throughout his admission, ranging from 200 mg/dL to 400 mg/dL. Subsequent testing also showed hemoglobin A1C (HbA1C) at 16.3%. The patient was discharged to home on hospital day 4 on a proper insulin regimen and choreiform symptom management with risperidone. At the time of discharge, his uncontrolled arm movement had decreased in frequency and intensity. Documentation from a follow-up appointment one month later noted that while his symptoms were still present, they were continuing to decrease in severity and frequency.

## Discussion

Non-ketotic hyperglycemia hemichorea-hemiballismus syndrome is a rare condition characterized by glucose levels greater than 200 mg/dL and continuous, uncontrollable, and irregular jerky movements localized to one side of the body [1]. This disease more commonly presents in females of East Asian descent with a mean age of onset of 71 years and an estimated prevalence of less than one in 100,000 [2]. It is more commonly seen in type 2 diabetics compared to type 1 diabetics [3].

Although the pathophysiology of non-ketotic hyperglycemia-induced chorea is not clearly understood, several presentations of this condition have been reported with antipsychotics, cerebrovascular damage, methamphetamine, decreased GABA levels, and acute ischemic stroke [2]. Typical clinical manifestations include a spectrum of involuntary, continuous movements on one side of the body, generally in the setting of diabetes [4]. The differential diagnosis for non-ketotic hyperglycemia hemichorea-hemiballismus syndrome may include neoplastic disorders of the brain, Huntington’s disease, stroke, trauma, or neurotoxic substance use [4].

The hypothesized mechanism involves a shift from aerobic to anaerobic metabolism during the pathogenesis of non-ketotic hyperglycemia, leading to decreased levels of GABA due to consumption, and subsequent disinhibition of the thalamus and hyperactivation of dopaminergic signaling, causing uncoordinated movements [3]. This is in comparison to diabetic ketoacidosis, where GABA substrates are synthesized, leading to regional metabolic imbalance and basal ganglia dysfunction. Poor glycemic control can lead to vascular complications beyond peripheral vascular disease, increase the risk of cerebrovascular ischemia, and lead to decreased cerebral blood flow. In general, for patients presenting with the clinical picture of sudden onset of uncontrolled movements, the initial workup should include neurological evaluation, as well as evaluation for non-ketotic hyperglycemia syndrome. MRI and serum glucose are both helpful in the prompt diagnosis of non-ketotic hyperglycemia hemichorea-hemiballismus syndrome. Insulin therapy that is administered quickly has been shown to reduce the time and severity of the involuntary movements.

## Conclusions

Non-ketotic hyperglycemia hemichorea-hemiballismus syndrome is a rare condition with unclear morbidity and mortality rates. Although this condition most commonly affects patients with underlying diabetes, it can also be the presenting symptom for patients who will be newly diagnosed with diabetes. The association of the writhing movements with other neurodegenerative disorders makes it even more difficult to diagnose this condition. Early recognition and treatment are important to give the greatest chance of motor recovery. Insulin therapy and other neuroleptic agents to support glycemic control and dopaminergic signaling are the mainstays of care. Proper long-term management of the patient’s diabetes and achieving adequate control of HbA1C in the long term is of great significance.

## References

[1] Pinsker JE, Shalileh K, Rooks VJ, Pinsker RW (2015). Hemichorea-hemiballism secondary to non-ketotic hyperglycemia. J Clin Med Res.

[2] Bendi VS, Matta A, Torres-Russotto D, Shou J (2018). Bilateral chorea/ballismus: detection and management of a rare complication of non-ketotic hyperglycaemia. BMJ Case Rep.

[3] Cosentino C, Torres L, Nuñez Y, Suarez R, Velez M, Flores M (2016). Hemichorea/hemiballism associated with hyperglycemia: report of 20 cases. Tremor Other Hyperkinet Mov (N Y).

[4] Bizet J, Cooper CJ, Quansah R, Rodriguez E, Teleb M, Hernandez GT (2014). Chorea, hyperglycemia, basal ganglia syndrome (C-H-BG) in an uncontrolled diabetic patient with normal glucose levels on presentation. Am J Case Rep.

